# Serum Actinin-4 Levels as a Potential Diagnostic and Prognostic Marker in Cervical Cancer

**DOI:** 10.1155/2020/5327378

**Published:** 2020-08-14

**Authors:** Xigui Ma, Huiying Xue, Jixiang Zhong, Bo Feng, Yanghua Zuo

**Affiliations:** ^1^Department of Traditional Chinese Medicine, Huai'an Maternal and Child Health Care Hospital, Renmin South Road 104, Huai'an 223002, China; ^2^Department of Reproductive Center, Huai'an Maternal and Child Health Care Hospital, Renmin South Road 104, Huai'an 223002, China

## Abstract

**Purpose:**

The present study was aimed at determining the serum levels of actinin-4 (ACTN4) in cervical cancer (CC) and investigating the diagnostic and prognostic value of serum ACTN4 in CC.

**Materials and Methods:**

We included 93 CC patients, 52 cervical intraepithelial neoplasia (CIN) patients, and 70 healthy women. Serum ACTN4 levels were assessed using an ELISA method. A receiver operating characteristic (ROC) curve was performed to evaluate the diagnostic value of serum ACTN4. The survival curves were used to display the overall survival distributions.

**Results:**

Serum ACTN4 levels in CC patients were 48.39 ± 13.98 pg/mL which is significantly higher than those in CIN patients (32.72 ± 9.44 pg/mL; *P* < 0.001) and those in healthy controls (30.84 ± 8.08 pg/mL; *P* < 0.001). The ROC analysis demonstrated that the area under the curve (AUC) of ACTN4 was 0.852 (95%CI = 0.796–0.908), with sensitivity of 76.3% and specificity of 87.7%. Serum ACTN4 levels were associated with the FIGO stage, lymph node metastasis, and lymphovascular space invasion of CC (all *P* < 0.05). The survival curve suggested that high serum ACTN4 levels were related to poor prognosis.

**Conclusion:**

Our findings suggest that serum ACTN4 levels may be valuable diagnostic and prognostic biomarkers for CC.

## 1. Introduction

Cervical cancer (CC) is the second most common female malignancy globally, and it is the most common female malignancy in developing countries which has high morbidity and mortality rates [1]. In recent years, the incidence of CC has increased greatly in young women under the age of 35 [2]. Despite great advances in surgical and adjuvant therapy, the overall survival of CC patients, especially that of advanced patients, is still very poor [3]. At present, a Pap smear combined with an HPV test has been used for the early screening of cervical lesions. However, the screening methods are invasive and costly, leading to lower screening coverage in China [3]. Previous studies have reported that the human papillomavirus (HPV) screening results have a relatively high false-positive rate and a relatively low specificity [4, 5]. In addition, the results of TCT interpretation by film-reading doctors are uneven, which might cause some misleadingness in the choices of prevention measures and treatment for CC [6]. Noteworthily, when applying the same treatment plan to patients with similar pathological types, the efficacy and prognosis are quite different. Therefore, it is necessary to identify new biomarkers directly related to the progression and prognosis of CC.

Alpha-actinins (ACTNs) are actin-binding proteins in the spectrin gene superfamily [7], which are known to be cross-linked with filamentous actin (F-actin) to maintain the integrity of cytoskeleton and to control cell motility [8]. The ACTN family has four members, numbered ACTN1–4, which are present in humans and other mammals [9–11]. ACTN4 is encoded by the ACTN4 gene and is widely expressed in many tissues, especially in glomerular podocytes [12]. ACTN4 has an actin-binding domain at the N-terminus, and ACTN4 monomers can form a homodimer through reverse binding, forming a dumbbell-shaped structure [13]. As an actin-binding protein, ACTN4 is closely related to enhancing cell viability and tumor invasion and metastasis [14]. Recent researches have reported that the expression of ACTN4 is significantly elevated in multiple cancers, including breast cancer [14], pancreatic cancer [15], ovarian cancer [16], and lung cancer [17]. In addition, the ACTN4 levels are markedly associated with the poor prognosis of lung cancer [18, 19], thyroid cancer [20], and salivary gland carcinoma [21]. An et al. [22] have found that the expression level of ACTN4 in human cervical tumors is dramatically higher than that in normal cervical tissues. Their finding demonstrated that ACTN4 promotes the epithelial-to-mesenchymal transition and tumorigenesis by regulating Snail expression and the Akt pathway in CC [22]. Therefore, the expression of ACTN4 in cervical tissues may be used in the clinical diagnosis and prognosis prediction of CC.

However, up to now, the significance of the serum ACTN4 levels in CC has not been evaluated. Hence, in the current study, the serum levels of ACTN4 in patients with CC were measured. In addition, we estimated the potential diagnostic and prognostic value of serum ACTN4 expression in CC.

## 2. Materials and Methods

### 2.1. Study Population

A retrospective study was designed to evaluate serum actinin-4 as a biomarker for CC. Between July 2012 and June 2014, 93 newly diagnosed female CC patients and 52 newly diagnosed female cervical intraepithelial neoplasia (CIN) patients who received treatment at Huai'an Maternal and Child Health Care Hospital (Huai'an, Jiangsu, China) were recruited. The diagnoses of all patients were verified by the histopathological examination. The patients with other types of tumor or autoimmune, atherosclerotic, and hematologic diseases were excluded. The mean age of CC patients was 47.3 years with a range of 26-78 years. Meanwhile, 70 healthy women with no evidence of neoplasms and other serious diseases were enrolled from the physical examination center in the same hospital. There was no significant difference in age among the CC, CIN, and healthy control groups. This study was consistent with the Helsinki declaration and was authorized by the Ethics Committee of Huai'an Maternal and Child Health Care Hospital (approval number: H20130504). All participants signed written informed consent.

### 2.2. Clinicopathologic Feature Collection and Follow-Up

By reviewing the medical records, we collected the clinicopathologic characteristics of the patients, including age at diagnosis, pathological type, FIGO stage, tumor differentiation, pelvic lymph node metastasis, tumor size, and lymphovascular space invasion. The CC patients were classified based on the revised FIGO staging system for CC in 2009. The tumor size was the maximum tumor diameter determined by a gynecologic oncologist during pelvic examination. The patients in stage 1A1 received hysterectomy; the patients in stages IB1 and IIB received radical hysterectomy and pelvic lymph node dissection; the patients with ≥stage IIB received radiotherapy or radiotherapy combined with chemotherapy. A regular telephone follow-up was conducted after treatment to obtain the overall survival (OS) time of CC patients, and the OS was defined as the time from diagnosis to death or the last follow-up. The follow-up was in accordance with the FIGO guidelines.

### 2.3. Blood Sample Collection and Detection of Serum Actinin-4 and SCCA

A 5 mL peripheral blood sample from each patient was collected before receiving any treatment. After standing at room temperature for 10 minutes, the blood samples were centrifugated at 1,500 g/min for 15 min, and then, the supernatant was stored at −80°C until further usage. The serum actinin-4 concentration was measured by a quantitative enzyme-linked immunosorbent assay (ELISA) method (Uscn Life Science Inc., Wuhan, China). The levels of SCCA in serum were determined using an ELISA kit (R&D Systems, Minneapolis, MN). The detection of all samples was strictly in accordance with the instructions provided by the manufacturer and was performed in duplicates.

### 2.4. Statistical Analysis

All statistical analyses were conducted by using SPSS 23.0 and GraphPad Prism 8. The continuous data following normal distribution were expressed as the mean ± standard deviation (SD). A *t*-test was used to compare serum ACTN4 levels between the two subgroups of each clinicopathological parameters, and the serum ACTN4 levels of CC patients, CIN patients, and healthy controls were compared by the SNK-*q* test. Receiver operating characteristic (ROC) curves were performed to assess the diagnostic value of serum ACTN4 levels for differentiating CC patients from CIN patients and healthy controls. The Kaplan-Meier method and log-rank test were used to plot survival curves. The Cox proportional hazards models in univariate and multivariate analyses were used for evaluating the prognostic value of serum ACTN4 expression. A two-tailed *P* value < 0.05 was considered to be statistically significant.

## 3. Results

### 3.1. Serum ACTN4 Levels Are Higher in Patients with CC

**Figure 1:**
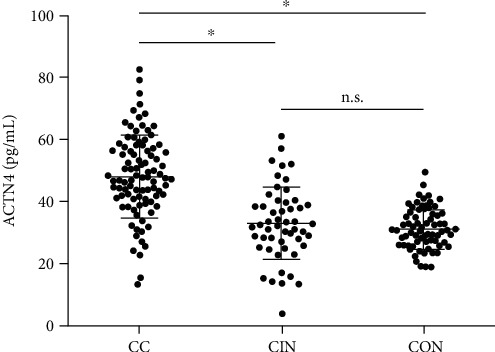
The serum ACTN4 levels in CC patients, CIN patients, and healthy controls. ^∗^*P* < 0.001.

Serum concentrations of ACTN4 were detected to range from 13.38 to 82.67 pg/mL with a mean (±SD) of 48.39 ± 13.98 pg/mL for CC patients, to range from 3.71 to 61.32 ng/mL with a mean (±SD) of 32.72 ± 9.44 pg/mL for CIN patients, and to range from 18.99 to 49.76 ng/mL with a mean (±SD) of 30.84 ± 8.08 pg/mL for healthy controls. Serum ACTN4 levels in CC patients were significantly higher than those in CIN patients and healthy controls (*P* < 0.001). However, no significant difference in serum ACTN4 was found between CIN patients and healthy controls (*P* = 0.607), as shown in Figure 1.

### 3.2. The Diagnostic Value of Serum ACTN4 Levels for CC

**Figure 2:**
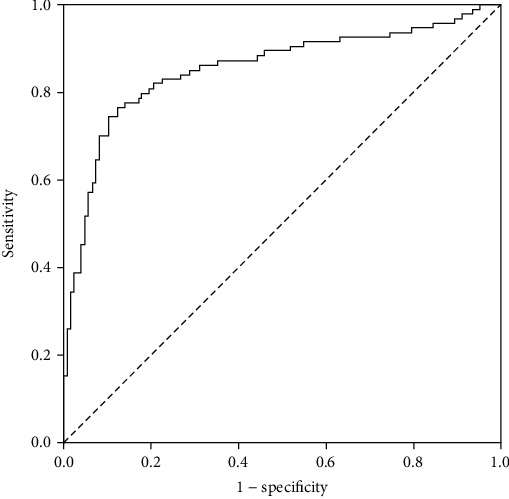
ROC curve analysis assessed the diagnostic performance of serum ACTN4 in CC. The AUC was 0.852, *P* < 0.001.

We next used ROC curve analysis to estimate the diagnostic value of serum ACTN4 expression for CC. The ROC curve showed that the serum levels of ACTN4 were robust for discriminating CC patients from benign and healthy control subjects, with an area under the curve (AUC) value of 0.852 (95%CI = 0.796–0.908), as demonstrated in Figure 2. According to maximum Youden's index, we used 40.62 pg/mL as the cutoff value, and the sensitivity and specificity were 76.3% and 87.7%, respectively.

### 3.3. Association between Serum ACTN4 Levels and Clinicopathological Parameters of CC Patients

We further investigated the correlations between serum levels of ACTN4 and clinical pathological data of 93 CC patients, and the results are demonstrated in Table 1. We observed that serum ACTN4 levels were related to the FIGO stage, lymph node metastasis, and lymphovascular space invasion (all *P* < 0.05). Nevertheless, no significant association was found between serum ACTN4 levels and age, pathological type, differentiation degree, and tumor size in CC patients (all *P* > 0.05).

### 3.4. Survival Analysis of Serum ACTN4 Levels in CC

**Figure 3:**
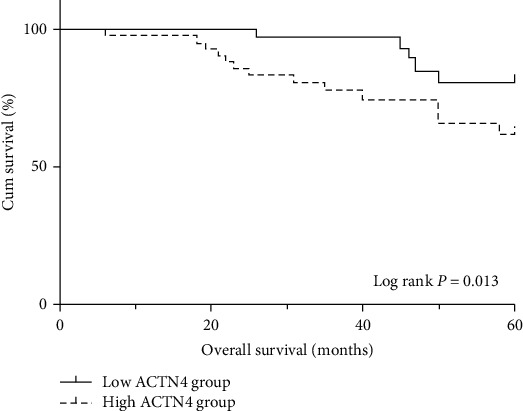
Kaplan-Meier curve compared OS of CC patients with high serum ACTN4 levels versus those with low serum ACTN4 levels.

During the follow-up period, nine CC patients were lost, and the followed up rate is 90.3%. Finally, the prognostic value of serum ACTN4 was assessed in 84 patients. The patients were followed up to December 2018. The range of follow-up time was 6 to 60 months, with the median time of 46.0 months and mean time of 43.2 months. According to the median serum levels of ACTN4 in CC patients (47.50 pg/mL), the 84 CC patients were divided into the high ACTN4 level group (<47.50 pg/mL, *N* = 42) and low ACTN4 level group (≥47.50 pg/mL, *N* = 42). The estimated 5-year OS of patients with high serum ACTN4 levels and low serum ACTN4 levels were 67.3% and 86.2%, respectively. The Kaplan-Meier survival curve and log-rank test indicated that CC patients with high serum ACTN4 levels had a worse prognosis than those with low serum ACTN4 levels (*P* = 0.013) (Figure 3).

Univariate Cox regression analyses showed that the serum ACTN4 levels (*P* < 0.001), FIGO stage (*P* < 0.001), differentiation degree (*P* = 0.012), lymph node metastasis (*P* < 0.001), and lymphovascular space invasion (*P* = 0.024) had significant prognostic value for OS. Multivariate analysis was further performed to evaluate the prognostic value of serum ACTN4 as an independent factor for CC. All the statistically significant factors from univariate analyses were included, and the results indicated that the FIGO stage and lymph node metastasis were the independent prognostic factors for CC (all *P* < 0.05) (Table 2).

## 4. Discussion

Cervical cancer is a heterogeneous disease with complicated etiology. Genetic and environmental factors play a crucial role in the pathogenesis of CC [23]. Although the diagnosis and prognosis of CC have improved greatly over the past few decades, it is necessary to improve early detection and screening methods to determine additional promising circulating biomarkers for better patient selection and more personalized treatments [24]. As far as we know, this study represented the first effort to evaluate the serum expression of ACTN4 as a new biomarker for CC.

As an actin-binding protein, ACTN4 can participate in regulating cell migration, invasion, and metastasis via regulating the actin filament flexibility at the leading edge of invading cancer cells [25, 26]. ACTN4-overexpressing cancer cells have the potential to metastasize, because the overexpression of ACTN4 protein in cancer cells can stimulate the dynamic reconstruction of the actin cytoskeleton [27]. Up to now, numerous studies have reported the association between ACTN4 and multiple cancers. Okamoto et al. [18] observed that ACTN4 is expressed in small-cell lung cancer (NSCLC), and it had a significant correlation with invasion and distant metastasis. Additionally, ACTN4 was reported to be a potential predictive biomarker for the efficacy of adjuvant chemotherapy in patients with NSCLC [19]. Watabe et al. [21] revealed that the copy number increase of ACTN4 is a novel indicator for poor overall survival of patients with salivary gland carcinoma, and the copy number variation would affect the expression of protein. A recent study demonstrated that serum ACTN4 levels were dramatically elevated in patients with breast cancer when compared to healthy controls, and serum ACTN4 may be an effective clinical indicator for diagnosing or predicting the clinical outcomes of breast cancer patients [14]. In addition, ACTN4 was proven to be associated with the pathogenesis of CC. An et al. [22] proposed a novel mechanism for epithelial-to-mesenchymal transition and tumorigenesis in CC which could be induced by ACTN4 through regulating Snail expression and *β*-catenin stabilization. Hence, it is significant to investigate the role of serum ACTN4 in CC.

In the current study, we observed that serum levels of ACTN4 in CC patients were statistically higher than those in CIN patients and those in healthy controls. However, serum ACTN4 levels were not significantly different between the CIN group and the control group. It was shown that serum ACTN4 expression could strongly differentiate CC patients from CIN patients and healthy controls. The ROC analysis demonstrated that the AUC of ACTN4 was 0.852, and at the optimal cutoff of 40.62 pg/mL, the sensitivity and specificity were, respectively, 76.3% and 87.7%, suggesting that serum ACTN4 might be a potential diagnostic biomarker for CC. In a recent study which included 800 Chinese women, Hu et al. [4] reported that the sensitivity and specificity of HPV screening in the diagnosis of CC were 77.25% and 65.37%. The sensitivity of the HPV test was a litter higher than that of serum ACTN4 detection, though the specificity of serum ACTN4 detection was well above that of the HPV test. Hence, comparing with the HPV test in diagnosing CC, detecting serum ACTN4 has some advantages. Furthermore, serum ACTN4 levels have been indicated to be a great biomarker for diagnosing multiple cancers. Fang et al. [14] in their study reported that serum ACTN4 was a promising indicator for diagnosing breast cancer, with the AUC of 0.887. Wang et al. [17] used ACTN4 expression in peripheral blood to differentiate NSCLC patients from healthy individuals in two groups of participants, and they obtained both satisfactory effects. Furthermore, we investigated the correlation between serum ACTN4 and clinical characteristics of CC patients. The serum ACTN4 levels were significantly associated with the FIGO stage, lymph node metastasis, and lymphovascular space invasion of CC, which suggests that ACTN4 could contribute to the development, invasion, and metastasis of CC. In addition, our results indicated that high ACTN4 levels were associated with the poor survival of CC patients. In the multivariate analysis, although ACTN4 levels did not reach the statistical significance, it still seems to be able to influence the OS.

However, several limitations in the present study should be taken into consideration. First, the sample size was relatively small, which was likely to reduce the statistical power of our results. Second, we only explored the relationship between serum ACTN4 and OS, and other prognostic indicators were not examined due to the incomplete data, which needs to be improved in the future. Third, this study was a primary study to determine the clinical significance of serum ACTN4 levels for the diagnosis and prognosis of CC, but the specific molecular mechanisms remain unclear. Hence, further experiments should be conducted to elucidate the mechanisms.

In conclusion, our study showed that serum ACTN4 levels were increased in CC patients and were related to the FIGO stage, lymph node metastasis, and lymphovascular space invasion of CC patients. In addition, serum levels of ACTN4 have great diagnostic and prognostic value in CC. Nevertheless, further studies with a larger sample size should be carried out to confirm our results.

## Figures and Tables

**Table 1:** Serum ACTN4 levels in CC patients according to clinicopathological parameters.

Parameters	*N*	%	ACTN4 (pg/mL)	*P*
Age (years)				
≤45	39	41.9	49.30 ± 12.36	0.491
>45	54	58.1	47.36 ± 13.93	
Pathological type				
Squamous cell carcinoma	72	77.4	47.66 ± 13.62	0.434
Adenocarcinoma	21	22.6	49.94 ± 12.07	
FIGO stage				
IA1-IB1	52	55.9	45.31 ± 12.67	0.017
≥IB2	41	44.1	52.29 ± 14.83	
Differentiation				
Well and moderately differentiated	63	67.7	47.38 ± 12.94	0.209
Poorly differentiated	30	32.3	50.65 ± 13.78	
Lymph node involvement				
Negative	66	71.0	43.65 ± 13.54	<0.001
Positive	27	29.0	59.97 ± 16.23	
Tumor size				
≤2	59	63.4	49.29 ± 12.67	0.284
>2	34	36.6	46.17 ± 14.19	
Lymphovascular space invasion				
Negative	58	62.3	44.21 ± 10.96	<0.001
Positive	35	37.6	55.64 ± 16.32	

**Table 2:** Univariate and multivariate Cox regression analysis of OS in CC patients.

Variables	Univariate			Multivariate		
	HR	95% CI	*P*	HR	95% CI	*P*
Age (>45 vs. ≤45 years)	1.331	0.712–2.372	0.643	—		
Pathological type (squamous cell carcinoma vs. adenocarcinoma)	1.106	0.903–1.341	0.874	—		
FIGO stage (≥IB2 vs. IA1-IB1)	2.818	1.746–4.112	<0.001	2.015	1.464–3.046	0.017
Differentiation (poorly differentiated vs. well and moderately differentiated)	1.874	1.412–3.648	0.012	1.593	1.198–2.749	0.156
Lymph node involvement (positive vs. negative)	4.621	2.815–7.492	<0.001	2.907	1.315–7.124	<0.001
Tumor size (>2 vs. ≤2 cm)	1.536	1.115–2.896	0.257	—		
Lymphovascular space invasion (positive vs. negative)	2.172	1.721–3.824	0.024	1.514	1.139–2.472	0.297
Serum ACTN4 levels (high vs. low levels)	2.442	1.806–4.113	<0.001	1.785	1.406–3.127	0.082

## Data Availability

The datasets used and/or analyzed during the present study are available from the corresponding author on reasonable request.
